# Virtual Strategies for the Broad Delivery of High Intensity Exercise in Persons With Spinal Cord Injury: Ongoing Studies and Considerations for Implementation

**DOI:** 10.3389/fspor.2021.703816

**Published:** 2021-08-06

**Authors:** David W. McMillan, Todd A. Astorino, Michael A. Correa, Mark S. Nash, David R. Gater

**Affiliations:** ^1^Christine E. Lynn Rehabilitation Center for the Miami Project to Cure Paralysis, UHealth/Jackson Memorial, Miami, FL, United States; ^2^Department of Physical Medicine & Rehabilitation, University of Miami Leonard M. Miller School of Medicine, Miami, FL, United States; ^3^Department of Kinesiology, California State University San Marcos, San Marcos, CA, United States; ^4^Department of Neurological Surgery, University of Miami Leonard M. Miller School of Medicine, Miami, FL, United States

**Keywords:** virtual fitness, home-based exercise, high intensity exercise, paralysis, disability

## Abstract

Spinal cord injury (SCI) results in a multitude of metabolic co-morbidities that can be managed by exercise. As in the non-injured population, manipulation of exercise intensity likely allows for fruitful optimization of exercise interventions targeting metabolic health in persons with SCI. In this population, interventions employing circuit resistance training (CRT) exhibit significant improvements in outcomes including cardiorespiratory fitness, muscular strength, and blood lipids, and recent exploration of high intensity interval training (HIIT) suggests the potential of this strategy to enhance health and fitness. However, the neurological consequences of SCI result in safety considerations and constrain exercise approaches, resulting in the need for specialized exercise practitioners. Furthermore, transportation challenges, inaccessibility of exercise facilities, and other barriers limit the translation of high intensity “real world” exercise strategies. Delivering exercise via online (“virtual”) platforms overcomes certain access barriers while allowing for broad distribution of high intensity exercise despite the limited number of population-specific exercise specialists. In this review, we initially discuss the need for “real world” high intensity exercise strategies in persons with SCI. We then consider the advantages and logistics of using virtual platforms to broadly deliver high intensity exercise in this population. Safety and risk mitigation are considered first followed by identifying strategies and technologies for delivery and monitoring of virtual high intensity exercise. Throughout the review, we discuss approaches from previous and ongoing trials and conclude by giving considerations for future efforts in this area.

## Introduction

Spinal cord injury (SCI) is an insult to the nervous systems that leads to a host of co-morbidities. For example, metabolic complications post-injury begin with a rapid and precipitous loss of lean muscle (Grimby et al., 1976; Castro et al., 1999; Gorgey and Dudley, 2007; Moore et al., 2015) and bone mass (Zleik et al., 2019; McMillan et al., 2021c) and increase in fat mass (Groah et al., 2011; Farkas et al., 2019; McCauley et al., 2020; Gater et al., 2021). The profound post-injury changes in body composition occur in parallel with disordered lipid (Brenes et al., 1986; Zlotolow et al., 1992; Karlsson et al., 1995; Maki et al., 1995; McGlinchey-Berroth et al., 1995; Nash et al., 2005; Emmons et al., 2010; Ellenbroek et al., 2014; La Fountaine et al., 2017, 2018) and glucose metabolism (Palmer et al., 1976; Duckworth et al., 1980, 1983; Karlsson et al., 1995; Aksnes et al., 1996; Bauman et al., 1999; Chilibeck et al., 1999; Jeon et al., 2002; Elder et al., 2004; Battram et al., 2007; Segal et al., 2007; Wang et al., 2009; Lewis et al., 2010; Gorgey and Gater, 2011; Yarar-Fisher et al., 2013). The coalition of these component risk factors is defined as cardiometabolic disease (CMD), and the Consortium for Spinal Cord Medicine has released Clinical Practice Guidelines for CMD in SCI (Nash et al., 2019). Importantly, these guidelines identify obesity as the most common CMD component risk factor in SCI, and other dyslipidemic CMD risk factors cluster in a manner favoring disordered fat metabolism after SCI (Libin et al., 2013). Mitigation strategies are warranted since this disordered fat metabolism in SCI is both a cause and the effect of the aberrant accumulation of whole-body fat mass, as well as regionally specific infiltration of fat deposits into various tissues such as skeletal muscle (Moore et al., 2015) and bone marrow (McMillan et al., 2021c). In persons without SCI, physical activity is an important management strategy for obesity (Piercy et al., 2018). Furthermore, regular physical activity is important for promoting health in SCI as well (Martin Ginis et al., 2018; Nash et al., 2019), but in this population there are considerations for tailoring exercise interventions to target specific outcomes such as obesity (McMillan et al., 2021b). Developed by experts in nutrition, exercise, rehabilitation, cardiology, endocrinology, and internal medicine, the Consortium's clinical practice guidelines (Nash et al., 2019) recommend exercise as a primary treatment for the management of CMD in SCI. These guidelines recommend “at least 150 min per week […] satisfied by sessions of 30–60 min performed three to five days per week, or by completing at least three 10-min sessions per day” (Nash et al., 2019). Furthermore, recent systematically developed (Appraisal of Guidelines, for Research, and Evaluation second edition; AGREE II) evidence-based activity guidelines developed by an international team of scientists endorse with moderate to high confidence the beneficial effects of exercise on CMD in persons with SCI (Martin Ginis et al., 2018). or These guidelines recommend “at least 30 min of moderate to vigorous intensity aerobic exercise three times per week” (Martin Ginis et al., 2018). Thus, current SCI CMD exercise guidelines are designed primarily around volume and frequency. However, experts are calling into question the importance of intensity in exercise prescription for persons with SCI (Nightingale et al., 2017a), especially in the context of addressing the metabolic co-morbidities of this condition.

In the general population, exercise intensity is an important parameter to consider for prescribing exercise to target CMD-related health outcomes (Weston et al., 2014; Batacan et al., 2017; Campbell et al., 2019; Taylor et al., 2019), with higher intensity exercise generally showing more favorable outcomes compared to moderate-intensity continuous training (MICT). Despite challenges related to proper implementation, there is a growing body of emerging evidence in SCI showing that higher exercise intensities promote greater physiological and potentially greater clinical benefits (Harnish C. et al., 2017; Nightingale et al., 2017a; Graham et al., 2019; McLeod et al., 2020), though not all results are universal (Solinsky et al., 2020). For example, multiple findings show the feasibility of high-intensity interval training (HIIT) arm cycling in SCI (de Groot et al., 2003; Harnish C. et al., 2017; Harnish C. R. et al., 2017; Nightingale et al., 2017a; Astorino and Thum, 2018b; Astorino, 2019; Graham et al., 2019; McLeod et al., 2020; McMillan et al., 2020; Solinsky et al., 2020), and one study demonstrates that HIIT performed on an arm ergometer allows a reduced time commitment to achieve a fixed calorie target vs. MICT (McMillan et al., 2020). However, all of these data stem from trials performed in laboratory settings in which the experimenter implements the exercise regimen and the only requirement of the patient is to follow his/her instructions. Moreover, one challenge of employing HIIT in a “real-world” setting is the staggering number of permutations of parameters including work and recovery durations and intensities. Real-time manipulation of these variables, especially while also exercising, can require substantial cognitive processing and experience with this modality that may be inappropriate for a novice exerciser to perform on their own. Other forms of intermittent exercise such as circuit resistance training (CRT) combining brief bouts of arm ergometry with dynamic whole-body resistance training have been employed in this population and show substantial metabolic effects (Jacobs et al., 2001, 2002; Nash et al., 2001, 2002, 2007; Kressler et al., 2014; McMillan et al., 2021a). Accordingly, a recent systematic review concluded that CRT elicits more favorable changes in the clinical lipid profile compared to aerobic exercise alone (Farrow et al., 2020). However, despite the emerging evidence supporting the role of exercise intensity in targeting CMD-related outcomes, there are many considerations for implementing “real world” high intensity exercise in this population.

Persons with SCI face multiple barriers to exercise participation and adherence, helping explain why this population spends such a high proportion (~87%) of the day in a sedentary state (Nightingale et al., 2017b). Internal barriers include lack of energy and motivation (Cowan et al., 2013). In addition, external barriers include factors such as transportation, the cost of exercise programs, the inaccessibility of fitness centers (Rimmer et al., 2000; Kehn and Kroll, 2009), and low trust in a non-specialized exercise instructor's ability to meet the unique needs of an individual with SCI (Rimmer et al., 2000; Scelza et al., 2005; Kehn and Kroll, 2009; Cowan et al., 2013). These barriers exceed the agency of the individual and are superimposed upon demographic variables such as socioeconomics, minority status, sex, age, and location of residence (Rimmer et al., 2000; Kehn and Kroll, 2009; Cowan et al., 2012). To address these population-specific barriers, recent studies have delivered exercise via online (“virtual”) platforms (Wilroy et al., 2017, 2020; Lai et al., 2019). Virtual strategies for delivering exercise to persons with SCI overcomes certain structural barriers while allowing for a limited number of specialists to broadly distribute population-specific exercise services to many exercisers regardless of geographic location. Although technological requirements for accessing virtual exercise platforms exclude certain people such as those without internet access, computer or smartphone, or lack of adequate hand function, the COVID-19 pandemic has caused a shift in internet usage in general (Lemenager et al., 2021) and toward virtual exercise participation in particular (Parker et al., 2021). This online transition presents an opportunity to broadly deliver high intensity exercise to persons with SCI, by using virtual platforms to deliver population-specific exercise safely and effectively to the people where they live.

### Explanation of Virtual Exercise

Virtual exercise (VEX) is any form of exercise that involves real-time exchange of information via the internet. As such, this practice mandatorily excludes a portion of the population that cannot or does not have online access. Researchers and practitioners must remain cognizant of the limitations to internet access. However, despite limitations in this domain of access, VEX overcomes barriers in other domains such as transportation and infrastructure by allowing exercise to be remotely delivered to participant's homes by professionals who have specific expertise regarding a given population's unique safety and implementation needs. While solutions to internet access may seem more feasible than upgrades to transportation and infrastructure, the former solution risks a greater potential for allowing the burden of responsibility to be shifted onto the individual whereas the later requires public resources. To mitigate this risk, we support a multifaceted approach including but extending beyond online services, and urge researchers and practitioners to share and mitigate the resource responsibility wherever possible.

There are various VEX delivery strategies available to SCI exercise providers. Simple unidirectional guidance can be provided via graphics on a web page or mobile app depicting exercises for participants to complete. More advanced unidirectional guidance can occur via pre-recorded “on demand” video of an exercise instructor conducting a follow-along exercise session for streaming at the participant's convenience. However, for neither of these options is real-time feedback or monitoring provided to the participant. Therefore, if desired and possible, multidirectional connection via video conferencing allows for instructor(s) and participant(s) to engage during the exercise session. This live streaming of VEX gives exercise professionals the potential to actively monitor participants during the session, allowing for remote delivery of exercise to specialty populations (Chen et al., 2018). The availability of virtual platforms has increased recently, especially since the online transition in response to the COVID-19 pandemic.

In April of 2021 we conducted an informal search of ClinicalTrials.gov to locate ongoing and current studies employing VEX in persons with SCI using the search terms: “spinal cord injury” AND (exercise OR “physical activity” OR workout) AND (online OR virtual OR “home based”). As of the search date, the search revealed five ongoing trials registered in the ClinicalTrials.gov system (NCT03024320, NCT04408287, NCT04564495, NCT03495986, and NCT04397250). Utilizing virtual platforms to deliver on-demand exercise instructions, in the form of graphics and/or pre-recorded videos, has been successfully implemented in adults with a mobility limitation including those with SCI as part of the “Scale Up Project Evaluating Responsiveness to Home Exercise and Lifestyle Tele-Health” (SUPER-HEALTH; NCT03024320) study (Wilroy et al., 2017, 2020; Lai et al., 2019; Rimmer et al., 2019). Further utilization of video conferencing technologies can be used to deliver an even more interactive service to exercise participants, similarly to the implementation of VEX for other populations (Hong et al., 2017; Chen et al., 2018). Currently, there are three ongoing registered trials involving real-time participant monitoring during home-based VEX individualized for persons with SCI. In the “Spinal Cord Injury Exercise and Nutrition Conceptual Engagement” (SCIENCE; NCT03495986) trial, a single participant conducting functional electrical stimulation (FES)-cycling exercise is monitored by the study team, with the study team having remote access to the FES ergometer. This trial requires months of FES exercise training and will determine its effects on body composition and other cardiometabolic health outcomes. In the “Improving Activity Engagement Among Persons with SCI During COVID-19” (NCT04408287) trial, a fitness instructor conducts real-time follow-along VEX sessions for a group of participants with SCI. In the “Home Based Tele-exercise for People with Chronic Neurological Impairments” (Telex; NCT04564495) trial, the effects of sessions of VEX delivered in real-time compared to pre-recorded sessions will be compared in adults with chronic neurological impairments including multiple sclerosis and SCI. The results of this trial will be pivotal in comparing the added benefit of having multidirectional exchange of information during VEX. The logistical cost of pre-recorded sessions is substantially less, greatly reducing the time requirements of the instructor. However, the added benefit of the real-time feedback from the instructor, as well as the interaction between and within the participants themselves, likely confers an added benefit not possible with pre-recorded sessions.

### Safety Considerations and Screening

The pathophysiology of SCI warrants unique considerations for VEX beyond the necessary adaptation of exercise mode to accommodate the altered volitional motor function of the participants. The participants and instructors need to aware of, and actively mitigate, the increased risk for autonomic dysreflexia (AD), hypotension due to circulatory hypokinesis (Hjeltnes, 1984), thermal dysregulation, skin injury, fracture (Jacobs and Nash, 2004), and musculoskeletal overuse injury (Vives Alvarado et al., 2021) consequent with SCI. Of greatest concern among exercising paraplegics and tetraplegics with SCI above the 5th thoracic vertebrae is AD (Karlsson, 1999) classified as an increase in systolic or diastolic blood pressure of >20 mmHg from baseline (Krassioukov et al., 2009). These transient episodes of uncoordinated sympathetic outflow occur 10 (Popok et al., 2017) to 40 (Hubli et al., 2015; West et al., 2015) times per day in response to peripheral stimuli. These episodes cause a rapid rise in blood pressure combined with either bradycardia or tachycardia and further symptoms such as sweating, goosebumps, headache, nausea, anxiety, and blurry vision (Karlsson, 1999; Krassioukov et al., 2009). It should be noted that due to a reduced blood pressure in higher levels of SCI (discussed below), AD-induced increases in blood pressure can be exploited during exercise known as “boosting” in athletes (Gee et al., 2015). However, boosting is not always intentional (Nightingale et al., 2021), influences exercise performance, and is considered dangerous and therefore is discouraged (Gee et al., 2015). Circulatory hypokinesis (Hjeltnes, 1984; Davis, 1993; Faghri et al., 2001; Dela et al., 2003) is a phenomenon of reduced cardiac output for any given oxygen uptake due to venous pooling in the paralyzed lower extremities during exercise. The outcome is possible hypokinetic hypotension during upper extremity volitional exercise (Hjeltnes, 1984; Davis, 1993) but not lower extremity electrically-stimulated exercise (Faghri et al., 2001; Dela et al., 2003) likely because active contraction transiently increases venous pressures (i.e., “skeletal muscle pump”). Along with skeletal muscle flaccidity, decentralization of the sympathetic efferent signals to blood vessels also contributes to the risk of hypokinetic hypotension because autonomic signals usually actively oppose vasodilation from local metabolites produced by skeletal muscle contraction (Dela et al., 2003). Autonomic decentralization of blood vessels and associated pseudomotor cholinergic efferents innervating sweat glands further results in an impaired ability to regulate body temperature during exercise (West et al., 2013), increasing risk for hyperthermia especially when environmental temperatures are high. The high intensity nature of HIIE requires a greater metabolic rate than MICE, possibly serving as a greater thermoregulatory challenge. Below the level of injury, risk of skin injury is significantly increased in response to abrasion and pressure changes that might occur due to excessive/enhanced movement required during more dynamic exercise such as HIIE or CRT. Bone loss begins very early post-injury (Edwards et al., 2014) with periarticular hip and knee bone mineral density decreasing 2 to 4% per month (Bieringsorensen et al., 1990; Edwards et al., 2014) and declining up to ~20% (Bauman et al., 2015; Goenka et al., 2018) within the first year of SCI, resulting in increased risk of fracture (Carbone et al., 2014; Gifre et al., 2014). These pathophysiological considerations are superimposed on the increased risk for musculoskeletal injury in SCI due to overuse (Bayley et al., 1987; Burnham et al., 1993; Curtis et al., 1999; Ballinger et al., 2000; Vives Alvarado et al., 2021) and possibly also spasticity (Hartkopp et al., 1998).

Due to these layered and intersecting risk factors, certain safety precautions should be undertaken when conducting VEX. A safety screening should be performed to establish a baseline and identify any absolute contraindications to exercise. Importantly, if an exercise candidate has any of the following, participation should be postponed until physician clearance is obtained: (1) uncontrolled AD, (2) history of syncope, syncope-like symptoms, or confirmed hypotension during exercise, (3) unhealed skin injury, (4) unhealed fracture, and/or (5) any of the general contraindications to exercise as outlined in the American College of Sports Medicine's Guidelines for Exercise Testing and Prescription (Liguori, 2020). Note that an AD event is technically defined as a 20–30 mmHg increase in systolic and/or diastolic arterial blood pressure (Krassioukov et al., 2009). Due to this, certain ongoing home-based exercise trials in persons with SCI (i.e., NCT03495986) employ remote monitoring of physiological responses to exercise. While this approach is preferable in higher risk situations, remote physiological monitoring is resource limited and thus symptom monitoring will be used in many contexts. The remote nature of VEX means that the participant and/or their attendant(s) will be required to independently respond in the event of an adverse event during an exercise session. Therefore, screening is important, and exercise instructors should be familiar with the population-specific risks as well as mitigation strategies. Participants should be reminded to empty the bladder before sessions and maintain a regular bowel program to avoid AD, maintain adequate hydration to reduce risk of hypotension, exercise in a temperature-controlled environment and not in direct sunlight to avoid hyperthermia, and be aware of proper equipment operation to avoid skin injury. In the case of participants who are deemed by their medical team to be at high risk for adverse response to VEX, active monitoring can be implemented. For example, in the aforementioned SCIENCE trial (NCT03495986), participants' heart rate, blood pressure, and oxygen-hemoglobin saturation are actively monitored by the study team during exercise. However, this resource-intensive approach is beyond the requirements for most persons with SCI to participate in VEX. Therefore, the equipment implemented to monitor these signs is going to vary depending on the individual risk of each VEX participant and should be determined by each participant's medical providers.

### Delivering Real World High Intensity Virtual Exercise

The Consortium's consensus-based clinical practice guidelines in the United States denote “at least 150 min per week […] satisfied by performing sessions of 30–60 min three to five days per week, or at least three 10-min sessions per day (Nash et al., 2019).” Other evidence-based physical activity guidelines for metabolic health persons with SCI have been updated to recommend “at least 30 min of moderate to vigorous intensity aerobic exercise three times per week (Martin Ginis et al., 2018).” These SCI activity guidelines are designed primarily around volume and frequency of activity with less clear recommendations for exercise intensity expressed according to %HR/VO_2_max or mode. The Exercise and Sport Science Australia (ESSA) position statement on exercise and SCI recommends “150 min/wk of moderate-intensity or 60 min/wk of vigorous-intensity” and allows for a range of intensities similar to recommendations for persons without SCI from governmental health authorities [e.g., CDC (Piercy et al., 2018) and WHO (Bull et al., 2020)]. It is important to understand that these intensity levels are relative, with average maximal rate of whole-body oxygen consumption in persons with tetraplegia (7.9–9.5 mk/kg/min) (Simmons et al., 2014) being below the values that qualify as “moderate” intensity (10.5–21.0 ml/kg/min) in persons without SCI (Piercy et al., 2018). The low rates of oxygen consumption and thus calorie expenditure that can be achieved and sustained during exercise by persons with SCI have important considerations for the tailoring of exercise to address various CMD-related outcomes (McMillan et al., 2021b), calling for novel approaches such as HIIT (Nightingale et al., 2017a) and CRT. These high intensity approaches have been shown in persons with SCI to increase post-exercise energy expenditure and fat utilization (McMillan et al., 2021a,b), thereby conferring benefits by influencing metabolism beyond the transient increases seen during exercise.

Despite the lack of emphasis in current SCI guidelines, experts have called for a recognition of the importance of exercise intensity in this population (Nightingale et al., 2017a). This call to “raise the intensity” originates from the important role that exercise intensity plays in the physiological responses and adaptations to exercise in persons without SCI. In adults without SCI, HIIT has classically been prescribed to enhance athletic performance (Billat, 2001) and more recently has been realized in the context of enhancing indices related to health status (Weston et al., 2014; Batacan et al., 2017; Campbell et al., 2019; Taylor et al., 2019). Notably, when using HIIT, a dramatically lower exercise volume is required to achieve increases in cardiorespiratory fitness and oxidative capacity compared to steady-state exercise (Burgomaster et al., 2008). Furthermore, HIIT enhances metabolic function thus more specifically addresses cardiometabolic disease component risks (Weston et al., 2014; Batacan et al., 2017; Campbell et al., 2019). Unfortunately, there is a paucity of research investigating efficacy of HIIT in persons with SCI, and the few HIIT interventions in SCI are limited by small sample size (de Groot et al., 2003; Harnish C. R. et al., 2017), subject heterogeneity, short training duration (Harnish C. et al., 2017), and complicated exercise modes (Solinsky et al., 2020). Studies examining the acute physiological response to HIIT (Astorino and Thum, 2018b; Astorino, 2019; Graham et al., 2019; McLeod et al., 2020; McMillan et al., 2020) in persons with SCI have established its safety and feasibility in persons with paraplegia, with preliminary safety and feasibility evidence indirectly available for tetraplegia (Solinsky et al., 2020). Importantly, the cardiovascular and metabolic response to exercise undulates with changing exercise intensity during a HIIT session in persons with SCI (McMillan et al., 2020), showing that SCI does not ablate the acute physiological responses that chronic adaptations are dependent upon. Furthermore, one study (McMillan et al., 2020) in persons with paraplegia demonstrated a reduced time commitment with HIIT vs. steady-state arm cycling to achieve a fixed energy expenditure equal to 120 kcal, providing indirect evidence for the time-efficiency of HIIT in SCI. In addition, results from one study in men and women with SCI (Astorino and Thum, 2018a) exhibited significantly higher enjoyment in response to submaximal or supramaximal interval exercise vs. continuous exercise, and no participants preferred the bout of continuous exercise. Gauthier et al. (2018) examined the feasibility and efficacy of six wk of home-based HIIT performed using their own wheelchair. Participants did not report any serious adverse events, deemed training to be feasible, and reported significant subjective improvements in health.

Despite this burgeoning evidence supporting HIIT in SCI, the broad delivery of high intensity exercise in this population has yet to be achieved. The use of VEX could allow for the widespread adoption of high intensity exercise by persons with SCI in a “real world” context. Accordingly, there are multiple on-going registered clinical trials deploying home-based high intensity exercise in persons with SCI. In the “High-intensity Interval Training for Cardiometabolic Health in Persons with Spinal Cord Injury” (NCT04397250) trial, high-intensity exercise sessions are completed at home with remote monitoring of cardiovascular responses (Farrow et al., 2021). In this study, practitioners administer arm cycles for home use, and prescribe a HIIT paradigm to determine the effect of a home-based HIIT intervention on cardiometabolic health outcomes. The “Telehealth High Intensity Interval Exercise and Cardiometabolic Health in Spinal Cord Injury” (Award Number R21NR019309) trial was recently funded, and while details about the trial have yet to be released, the purpose is to evaluate the effect of a “home-based telehealth HIIT arm crank exercise training program” on cardiometabolic health outcomes. These trials will be the first to scientifically test the use of VEX to deliver high intensity exercise to persons with SCI. However, it should be noted that at least two previous studies have demonstrated the feasibility of delivering non-virtual home-based high intensity exercise in persons with SCI (Nash et al., 2002; Solinsky et al., 2020). One of these studies used FES-assisted rowing (Solinsky et al., 2020), a complicated mode of exercise that is not readily accessible in a “real world” context. The other study (Nash et al., 2002); however, employed a modified version of a well-established CRT paradigm (Jacobs et al., 2001, 2002; Nash et al., 2001, 2002, 2007; Kressler et al., 2014; McMillan et al., 2021a) that has been shown to benefit multiple components of fitness and health in persons with SCI (Jacobs et al., 2001, 2002; Nash et al., 2001, 2007; Kressler et al., 2014). In this study, simple and inexpensive adaptations—such as using resistance bands attached to a home door—were used to reproduce the CRT in a home environment, and similar cardiovascular and metabolic responses were shown with the home-based vs gym-based CRT variants (Nash et al., 2001). Resourceful approaches such as this can now be combined with increases in internet access to broadly deliver VEX in a “real world” context to persons with SCI. This combination will possibly influence the viability of high intensity VEX in persons with SCI (Astorino et al., 2021), especially given the known (e.g., Astorino and Thum, 2018a) and anticipated psychological and social impacts of HIIT in SCI. For example, it has been demonstrated that HIIT is more enjoyable than MICT in persons with SCI (Astorino and Thum, 2018a). This increased enjoyment could yeild a benefit to both recruitment and retention. Additionally, intrapersonal as well as interpersonal incentives should be considered. For example, exercise professionals can capitalize on the social exchange that can occur during VEX. Recent qualitative evidence showed that persons with SCI desire to belong to a collective of SCI peers when participating in VEX (Lai et al., 2021). As suggested by research demonstrating associations between exercise and social belonging (Ginis et al., 2010; Richardson et al., 2017), integrating prosocial components into VEX could prove beneficial to uptake, adherence, and impact.

### Conclusions

Exercise's health benefits have been recognized since ancient times (Booth et al., 2002), but advances in technology allow us to reconceptualize the means by which we deliver this potent biological stimulus. The rising tide of digital technology will result in a merging of uni- and multi-directional platforms, allowing for the flexibility of on-demand content and the granularity and interactivity of real-time sessions. After accounting for population-specific safety considerations, this VEX will allow for persons with SCI to connect with specialized exercise practitioners without specific transportation and infrastructure requirements. Overcoming these barriers *via* VEX allows for increased access to specialized exercise required to optimize function and health in persons with SCI. Emerging evidence supports a call to “raise the intensity” in SCI, while the COVID-19 pandemic has increased internet usage and our familiarity with virtual platforms facilitating social exchange. When taken together, now is the time to employ VEX for the broad delivery of “real world” high intensity exercise in persons with SCI.

## Author Contributions

TA and DM conceptualized the topic of this manuscript. DM and MC developed the outline and first draft for the manuscript. All authors contributed edits that yielded the final form of the manuscript.

## Conflict of Interest

The authors declare that the research was conducted in the absence of any commercial or financial relationships that could be construed as a potential conflict of interest.

## Publisher's Note

All claims expressed in this article are solely those of the authors and do not necessarily represent those of their affiliated organizations, or those of the publisher, the editors and the reviewers. Any product that may be evaluated in this article, or claim that may be made by its manufacturer, is not guaranteed or endorsed by the publisher.

## References

[B1] Aksnes A. K. Hjeltnes N. Wahlstrom E. O. Katz A. Zierath J. R. Wallberg-Henriksson H. (1996). Intact glucose transport in morphologically altered denervated skeletal muscle from quadriplegic patients. Am. J. Physiol. 271(3 Pt 1), E593–E600. 10.1152/ajpendo.1996.271.3.E593 8843756

[B2] Astorino T. A. (2019). Hemodynamic and cardiorespiratory responses to various arm cycling regimens in men with spinal cord injury. Spinal Cord Ser. Cases 5:8. 10.1038/s41394-018-0145-9 30675386 PMC6333790

[B3] Astorino T. A. Hicks A. L. Bilzon J. L. J. (2021). Viability of high intensity interval training in persons with spinal cord injury-a perspective review. Spinal Cord 59, 3–8. 10.1038/s41393-020-0492-9 32483336

[B4] Astorino T. A. Thum J. S. (2018a). Interval training elicits higher enjoyment versus moderate exercise in persons with spinal cord injury. J. Spinal Cord Med. 41, 77–84. 10.1080/10790268.2016.1235754 27808004 PMC5810810

[B5] Astorino T. A. Thum J. S. (2018b). Within-session responses to high-intensity interval training in spinal cord injury. Disabil Rehabil. 40, 444–449. 10.1080/09638288.2016.1260648 27930890

[B6] Ballinger D. A. Rintala D. H. Hart K. A. (2000). The relation of shoulder pain and range-of-motion problems to functional limitations, disability, and perceived health of men with spinal cord injury: a multifaceted longitudinal study. Arch. Phys. Med. Rehabil. 81, 1575–1581. 10.1053/apmr.2000.18216 11128892

[B7] Batacan R. B. Jr. Duncan M. J. Dalbo V. J. Tucker P. S. Fenning A. S. (2017). Effects of high-intensity interval training on cardiometabolic health: a systematic review and meta-analysis of intervention studies. Br. J. Sports Med. 51, 494–503. 10.1136/bjsports-2015-095841 27797726

[B8] Battram D. S. Bugaresti J. Gusba J. Graham T. E. (2007). Acute caffeine ingestion does not impair glucose tolerance in persons with tetraplegia. J. Appl. Physiol. 102, 374–381. 10.1152/japplphysiol.00901.2006 17068214

[B9] Bauman W. A. Adkins R. H. Spungen A. M. Waters R. L. (1999). The effect of residual neurological deficit on oral glucose tolerance in persons with chronic spinal cord injury. Spinal Cord 37, 765–771. 10.1038/sj.sc.3100893 10578247

[B10] Bauman W. A. Cirnigliaro C. M. La Fountaine M. F. Martinez L. Kirshblum S. C. Spungen A. M. (2015). Zoledronic acid administration failed to prevent bone loss at the knee in persons with acute spinal cord injury: an observational cohort study. J. Bone Miner. Metab. 33, 410–421. 10.1007/s00774-014-0602-x 25158630

[B11] Bayley J. C. Cochran T. Sledge C. (1987). The weight-bearing shoulder. The impingement syndrome in paraplegics. J. Bone Joint Surg. Am. 69, 676–678. 10.2106/00004623-198769050-00006 3597466

[B12] Bieringsorensen F. Bohr H. H. Schaadt O. P. (1990). Longitudinal-study of bone-mineral content in the lumbar spine, the forearm and the lower-extremities after spinal-cord injury. Eur. J. Clin. Invest. 20, 330–335. 10.1111/j.1365-2362.1990.tb01865.x 2114994

[B13] Billat L. V. (2001). Interval training for performance: a scientific and empirical practice - special recommendations for middle- and long-distance running, part I: aerobic interval training. Sports Med. 31, 13–31. 10.2165/00007256-200131010-00002 11219499

[B14] Booth F. W. Chakravarthy M. V. Gordon S. E. Spangenburg E. E. (2002). Waging war on physical inactivity: using modern molecular ammunition against an ancient enemy. J. Appl. Physiol. 93, 3–30. 10.1152/japplphysiol.00073.2002 12070181

[B15] Brenes G. Dearwater S. Shapera R. LaPorte R. E. Collins E. (1986). High density lipoprotein cholesterol concentrations in physically active and sedentary spinal cord injured patients. Arch. Phys. Med. Rehabil. 67, 445–450. 3729689

[B16] Bull F. C. Al-Ansari S. S. Biddle S. Borodulin K. Buman M. P. Cardon G.. (2020). World Health Organization 2020 guidelines on physical activity and sedentary behaviour. Br. J. Sports Med. 54, 1451–1462. 10.1136/bjsports-2020-102955 33239350 PMC7719906

[B17] Burgomaster K. A. Howarth K. R. Phillips S. M. Rakobowchuk M. MacDonald M. J. Mcgee S. L.. (2008). Similar metabolic adaptations during exercise after low volume sprint interval and traditional endurance training in humans. J. Physiol. -London 586, 151–160. 10.1113/jphysiol.2007.142109 17991697 PMC2375551

[B18] Burnham R. S. May L. Nelson E. Steadward R. Reid D. C. (1993). Shoulder pain in wheelchair athletes: the role of muscle imbalance. Am. J. Sports Med. 21, 238–242. 10.1177/036354659302100213 8465919

[B19] Campbell W. W. Kraus W. E. Powell K. E. Haskell W. L. Janz K. F. Jakicic J. M.. (2019). High-intensity interval training for cardiometabolic disease prevention. Med. Sci. Sports Exerc. 51, 1220–1226. 10.1249/MSS.0000000000001934 31095079 PMC6777577

[B20] Carbone L. D. Chin A. S. Burns S. P. Svircev J. N. Hoenig H. Heggeness M.. (2014). Mortality after lower extremity fractures in men with spinal cord injury. J. Bone Miner. Res. 29, 432–439. 10.1002/jbmr.2050 23873733

[B21] Castro M. J. Apple D. F. Jr. Hillegass E. A. Dudley G. A. (1999). Influence of complete spinal cord injury on skeletal muscle cross-sectional area within the first 6 months of injury. Eur. J. Appl. Physiol. Occup. Physiol. 80, 373–378. 10.1007/s004210050606 10483809

[B22] Chen J. J. Cooper D. M. Haddad F. Sladkey A. Nussbaum E. Radom-Aizik S. (2018). Tele-exercise as a promising tool to promote exercise in children with cystic fibrosis. Front. Public Health 6:269. 10.3389/fpubh.2018.00269 30324099 PMC6172297

[B23] Chilibeck P. D. Bell G. Jeon J. Weiss C. B. Murdoch G. MacLean I.. (1999). Functional electrical stimulation exercise increases GLUT-1 and GLUT-4 in paralyzed skeletal muscle. Metabolism 48, 1409–1413. 10.1016/S0026-0495(99)90151-8 10582549

[B24] Cowan R. Nash M. Anderson-Erisman K. (2012). Perceived exercise barriers and odds of exercise participation among persons with SCI living in high-income households. Top. Spinal Cord Inj. Rehabil. 18, 126–127. 10.1310/sci1802-126 23459672 PMC3584768

[B25] Cowan R. E. Nash M. S. Anderson K. D. (2013). Exercise participation barrier prevalence and association with exercise participation status in individuals with spinal cord injury. Spinal Cord 51, 27–32. 10.1038/sc.2012.53 22584283

[B26] Curtis K. A. Drysdale G. A. Lanza R. D. Kolber M. Vitolo R. S. West R. (1999). Shoulder pain in wheelchair users with tetraplegia and paraplegia. Arch. Phys. Med. Rehabil. 80, 453–457. 10.1016/S0003-9993(99)90285-X 10206610

[B27] Davis G. M. (1993). Exercise capacity of individuals with paraplegia. Med. Sci. Sports Exerc. 25, 423–432. 10.1249/00005768-199304000-00004 8479296

[B28] de Groot P. C. Hjeltnes N. Heijboer A. C. Stal W. Birkeland K. (2003). Effect of training intensity on physical capacity, lipid profile and insulin sensitivity in early rehabilitation of spinal cord injured individuals. Spinal Cord. 41, 673–679. 10.1038/sj.sc.3101534 14639446

[B29] Dela F. Mohr T. Jensen C. M. R. Haahr H. L. Secher N. H. Biering-Sorensen F.. (2003). Cardiovascular control during exercise - insights from spinal cord-injured humans. Circulation 107, 2127–2133. 10.1161/01.CIR.0000065225.18093.E4 12695298

[B30] Duckworth W. C. Jallepalli P. Solomon S. S. (1983). Glucose-intolerance in spinal-cord injury. Arch. Phys. Med. Rehabil. 64, 107–110. 6338859

[B31] Duckworth W. C. Solomon S. S. Jallepalli P. Heckemeyer C. Finnern J. Powers A. (1980). Glucose-intolerance due to insulin resistance in patients with spinal-cord injuries. Diabetes 29, 906–910. 10.2337/diab.29.11.906 7429029

[B32] Edwards W. B. Schnitzer T. J. Troy K. L. (2014). Reduction in proximal femoral strength in patients with acute spinal cord injury. J. Bone Miner. Res. 29, 2074–2079. 10.1002/jbmr.2227 24677293

[B33] Elder C. P. Apple D. F. Bickel C. S. Meyer R. A. Dudley G. A. (2004). Intramuscular fat and glucose tolerance after spinal cord injury - a cross-sectional study. Spinal Cord 42, 711–776. 10.1038/sj.sc.3101652 15303112

[B34] Ellenbroek D. Kressler J. Cowan R. E. Burns P. A. Mendez A. J. Nash M. S. (2014). Effects of prandial challenge on triglyceridemia, glycemia, and pro-inflammatory activity in persons with chronic paraplegia. J. Spinal Cord Med. 10.1179/2045772314Y.0000000199 24617559 PMC4612202

[B35] Emmons R. R. Garber C. E. Cirnigliaro C. M. Moyer J. M. Kirshblum S. C. Galea M. D.. (2010). The influence of visceral fat on the postprandial lipemic response in men with paraplegia. J. Am. Coll. Nutr. 29, 476–481. 10.1080/07315724.2010.10719884 21504974

[B36] Faghri P. D. Yount J. P. Pesce W. J. Seetharama S. Votto J. J. (2001). Circulatory hypokinesis and functional electric stimulation during standing in persons with spinal cord injury. Arch. Phys. Med. Rehabil. 82, 1587–1595. 10.1053/apmr.2001.25984 11689980

[B37] Farkas G. J. Gorgey A. S. Dolbow D. R. Berg A. S. Gater D. R. (2019). Sex dimorphism in the distribution of adipose tissue and its influence on proinflammatory adipokines and cardiometabolic profiles in motor complete spinal cord injury. J. Spinal Cord Med. 42, 430–436. 10.1080/10790268.2018.1436125 29465306 PMC6718133

[B38] Farrow M. Nightingale T. E. Maher J. McKay C. D. Thompson D. Bilzon J. (2020). The effect of exercise on cardiometabolic risk factors in adults with chronic spinal cord injury: a systematic review. Arch. Phys. Med. Rehabil. 101, 2177–2205. 10.1016/j.apmr.2020.04.020 32445849

[B39] Farrow M. T. Maher J. Thompson D. Bilzon J. L. J. (2021). Effect of high-intensity interval training on cardiometabolic component risks in persons with paraplegia: Protocol for a randomized controlled trial. Exp Physiol. 106, 1159–1165. 10.1113/EP089110 33600014

[B40] Gater D. R. Jr. Farkas G. J. Tiozzo E. (2021). Pathophysiology of neurogenic obesity after spinal cord injury. Top. Spinal Cord Inj. Rehabil. 27, 1–10. 10.46292/sci20-00067 33814879 PMC7983633

[B41] Gauthier C. Brosseau R. Hicks A. L. Gagnon D. H. (2018). Feasibility, safety, and preliminary effectiveness of a home-based self-managed high-intensity interval training program offered to long-term manual wheelchair users. Rehabil. Res. Pract. 2018:8209360. 10.1155/2018/8209360 29888007 PMC5985105

[B42] Gee C. M. West C. R. Krassioukov A. V. (2015). Boosting in elite athletes with spinal cord injury: a critical review of physiology and testing procedures. Sports Med. 45, 1133–1142. 10.1007/s40279-015-0340-9 26009300

[B43] Gifre L. Vidal J. Carrasco J. Portell E. Puig J. Monegal A.. (2014). Incidence of skeletal fractures after traumatic spinal cord injury: a 10-year follow-up study. Clin. Rehabil. 28, 361–369. 10.1177/0269215513501905 24096543

[B44] Ginis K. M. Jetha A. Mack D. E. Hetz S. (2010). Physical activity and subjective well-being among people with spinal cord injury: a meta-analysis. Spinal Cord 48, 65–72. 10.1038/sc.2009.87 19581918

[B45] Goenka S. Sethi S. Pandey N. Joshi M. Jindal R. (2018). Effect of early treatment with zoledronic acid on prevention of bone loss in patients with acute spinal cord injury: a randomized controlled trial. Spinal Cord 56, 1207–1211. 10.1038/s41393-018-0195-7 30258212

[B46] Gorgey A. S. Dudley G. A. (2007). Skeletal muscle atrophy and increased intramuscular fat after incomplete spinal cord injury. Spinal Cord 45, 304–319. 10.1038/sj.sc.3101968 16940987

[B47] Gorgey A. S. Gater D. R. (2011). A preliminary report on the effects of the level of spinal cord injury on the association between central adiposity and metabolic profile. PM R. 3, 440–446. 10.1016/j.pmrj.2011.01.011 21570032

[B48] Graham K. Yarar-Fisher C. Li J. McCully K. M. Rimmer J. H. Powell D.. (2019). Effects of high-intensity interval training versus moderate-intensity training on cardiometabolic health markers in individuals with spinal cord injury: a pilot study. Top. Spinal Cord Inj. Rehabil. 25, 248–259. 10.1310/sci19-00042 31548792 PMC6743747

[B49] Grimby G. Broberg C. Krotkiewska I. Krotkiewski M. (1976). Muscle fiber composition in patients with traumatic cord lesion. Scand. J. Rehabil. Med. 8, 37–42. 132700

[B50] Groah S. L. Nash M. S. Ward E. A. Libin A. Mendez A. J. Burns P.. (2011). Cardiometabolic risk in community-dwelling persons with chronic spinal cord injury. J. Cardiopulm. Rehabil. Prev. 31, 73–80. 10.1097/HCR.0b013e3181f68aba 21045711

[B51] Harnish C. Sabo R. Daniels J. Caruso D. (2017). The effects of two weeks of arm crank sprint interval training in men with chronic spinal cord injury. Int. J. Sports Exerc. Med. 3, 56–59. 10.23937/2469-5718/1510059

[B52] Harnish C. R. Daniels J. A. Caruso D. (2017). Training response to high-intensity interval training in a 42-year-old man with chronic spinal cord injury. J. Spinal Cord Med. 40, 246–249. 10.1080/10790268.2015.1136783 26781683 PMC5430483

[B53] Hartkopp A. Murphy R. L. Mohr T. Kjcer M. Biering-Sorensen F. (1998). Bone fracture during electrical stimulation of the quadriceps in a spinal cord injured subject. Arch. Phys. Med. Rehabil. 79, 1133–1136. 10.1016/S0003-9993(98)90184-8 9749697

[B54] Hjeltnes H. (1984). Control of medical rehabilitation of para-and tetraplegics by repeated evaluation of endurance capacity. Int. J. Sports Med. 5, 171–174. 10.1055/s-2008-1025991

[B55] Hong J. Kim J. Kim S. W. Kong H.-J. (2017). Effects of home-based tele-exercise on sarcopenia among community-dwelling elderly adults: body composition and functional fitness. Exp. Gerontol. 87, 33–39. 10.1016/j.exger.2016.11.002 27838369

[B56] Hubli M. Gee C. M. Krassioukov A. V. (2015). Refined assessment of blood pressure instability after spinal cord injury. Am. J. Hypertens. 28, 173–181. 10.1093/ajh/hpu122 24990527

[B57] Jacobs K. A. Mahoney E. T. Nash M. S. Green B. A. (2002). Circuit resistance training in persons with complete paraplegia. J. Rehabil. Res. Dev. 39, 21–28. 11926325

[B58] Jacobs P. L. Nash M. S. (2004). Exercise recommendations for individuals with spinal cord injury. Sports Med. 34, 727–751. 10.2165/00007256-200434110-00003 15456347

[B59] Jacobs P. L. Nash M. S. Rusinowski J. W. (2001). Circuit training provides cardiorespiratory and strength benefits in persons with paraplegia. Med. Sci. Sports Exerc. 33, 711–717. 10.1097/00005768-200105000-00005 11323537

[B60] Jeon J. Y. Weiss C. B. Steadward R. D. Ryan E. Burnham R. S. Bell G. (2002). Improved glucose tolerance and insulin sensitivity after electrical stimulation-assisted cycling in people with spinal cord injury. Spinal Cord 40, 110–117. 10.1038/sj.sc.3101260 11859437

[B61] Karlsson A. K. Attvall S. Jansson P. A. Sullivan L. Lonnroth P. (1995). Influence of the sympathetic nervous system on insulin sensitivity and adipose tissue metabolism: a study in spinal cord-injured subjects. Metabolism 44, 52–58. 10.1016/0026-0495(95)90289-9 7854166

[B62] Karlsson A.K. (1999). Autonomic dysreflexia. Spinal Cord 37, 383–391. 10.1038/sj.sc.3100867 10432257

[B63] Kehn M. Kroll T. (2009). Staying physically active after spinal cord injury: a qualitative exploration of barriers and facilitators to exercise participation. BMC Public Health 9:168. 10.1186/1471-2458-9-168 19486521 PMC2694784

[B64] Krassioukov A. Warburton D. E. Teasell R. Eng J. J. Team S. C. I. R. E. R. (2009). A systematic review of the management of autonomic dysreflexia after spinal cord injury. Arch. Phys. Med. Rehabil. 90, 682–695. 10.1016/j.apmr.2008.10.017 19345787 PMC3108991

[B65] Kressler J. Burns P. A. Betancourt L. Nash M. S. (2014). Circuit training and protein supplementation in persons with chronic tetraplegia. Med. Sci. Sports Exerc. 46, 1277–1284. 10.1249/MSS.0000000000000250 24389521

[B66] La Fountaine M. F. Cirnigliaro C. M. Hobson J. C. Dyson-Hudson T. A. Mc Kenna C. Kirshblum S. C.. (2018). Establishing a threshold to predict risk of cardiovascular disease from the serum triglyceride and high-density lipoprotein concentrations in persons with spinal cord injury. Spinal Cord 56, 1051–1058. 10.1038/s41393-018-0187-7 30089895 PMC6219899

[B67] La Fountaine M. F. Cirnigliaro C. M. Kirshblum S. C. McKenna C. Bauman W. A. (2017). Effect of functional sympathetic nervous system impairment of the liver and abdominal visceral adipose tissue on circulating triglyceride-rich lipoproteins. PLoS ONE 12:e0173934. 10.1371/journal.pone.0173934 28346471 PMC5367791

[B68] Lai B. Wilroy J. Young H.-J. Howell J. Rimmer J. H. Mehta T.. (2019). A mobile app to promote adapted exercise and social networking for people with physical disabilities: usability study. JMIR Form. Res. 3:e11689. 10.2196/11689 30888325 PMC6444218

[B69] Lai B. W. Rimmer J. H. Yates A. Jeter A. Young H.-J. Thirumalai M.. (2021). Critical factors influencing the decision to enroll in a physical activity intervention among a predominant group of adults with spinal cord injury: a grounded theory study. Spinal Cord 59, 17–25. 10.1038/s41393-020-0530-7 32747672 PMC7397960

[B70] Lemenager T. Neissner M. Koopmann A. Reinhard I. Georgiadou E. Muller A.. (2021). COVID-19 lockdown restrictions and online media consumption in Germany. Int. J. Env. Res. Pub. He. 18:14. 10.3390/ijerph18010014 33375139 PMC7792961

[B71] Lewis J. G. Jones L. M. Legge M. Elder P. A. (2010). Corticosteroid-binding globulin, cortisol, free cortisol, and sex hormone-binding globulin responses following oral glucose challenge in spinal cord-injured and able-bodied men. Horm. Metab. Res. 42, 882–886. 10.1055/s-0030-1265128 20839151

[B72] Libin A. Tinsley E. A. Nash M. S. Mendez A. J. Burns P. Elrod M.. (2013). Cardiometabolic risk clustering in spinal cord injury: results of exploratory factor analysis. Top. Spinal Cord Inj. Rehabil. 19, 183–194. 10.1310/sci1903-183 23960702 PMC3743968

[B73] Liguori G. (2020). Medicine ACoS. ACSM's Guidelines for Exercise Testing and Prescription. Lippincott: Williams & Wilkins.

[B74] Maki K. C. Briones E. R. Langbein W. E. Inman-Felton A. Nemchausky B. Welch M.. (1995). Associations between serum lipids and indicators of adiposity in men with spinal cord injury. Paraplegia 33, 102–109. 10.1038/sc.1995.24 7753565

[B75] Martin Ginis K. A. van der Scheer J. W. Latimer-Cheung A. E. Barrow A. Bourne C. Carruthers P.. (2018). Evidence-based scientific exercise guidelines for adults with spinal cord injury: an update and a new guideline. Spinal Cord 56, 308–321. 10.1038/s41393-017-0017-3 29070812

[B76] McCauley L. S. Ghatas M. P. Sumrell R. M. Cirnigliaro C. M. Kirshblum S. C. Bauman W. A.. (2020). Measurement of visceral adipose tissue in persons with spinal cord injury by magnetic resonance imaging and dual x-ray absorptiometry: generation and application of a predictive equation. J. Clin. Densitom. 23, 63–72. 10.1016/j.jocd.2018.12.003 30638769

[B77] McGlinchey-Berroth R. Morrow L. Ahlquist M. Sarkarati M. Minaker K. L. (1995). Late-life spinal cord injury and aging with a long term injury: characteristics of two emerging populations. J. Spinal Cord Med. 18, 183–193. 10.1080/10790268.1995.11719391 7552423

[B78] McLeod J. C. Diana H. Hicks A. L. (2020). Sprint interval training versus moderate-intensity continuous training during inpatient rehabilitation after spinal cord injury: a randomized trial. Spinal Cord 58, 106–115. 10.1038/s41393-019-0345-6 31462757

[B79] McMillan D. W. Kressler J. Jacobs K. A. Nash M. S. (2021a). Substrate metabolism during recovery from circuit resistance exercise in persons with spinal cord injury. Eur. J. Appl. Physiol. 121, 1631–1640. 10.1007/s00421-021-04629-0 33655367

[B80] McMillan D. W. Maher J. L. Jacobs K. A. Nash M. S. Bilzon J. L. (2020). Physiological responses to moderate intensity continuous and high-intensity interval exercise in persons with paraplegia. Spinal Cord 1–8. 10.1038/s41393-020-0520-9 32681118

[B81] McMillan D. W. Maher J. L. Jacobs K. A. Nash M. S. Gater D. R. Jr. (2021b). Exercise interventions targeting obesity in persons with spinal cord injury. Top. Spinal Cord Inj. Rehabil. 27, 109–120. 10.46292/sci20-00058 33814889 PMC7983638

[B82] McMillan D. W. Nash M. S. Gater D. R. Jr Valderrábano R. J. (2021c). Neurogenic obesity and skeletal pathology in spinal cord injury. Top. Spinal Cord Inj. Rehabil. 27, 57–67. 10.46292/sci20-00035 33814883 PMC7983641

[B83] Moore C. D. Craven B. C. Thabane L. Laing A. C. Frank-Wilson A. W. Kontulainen S. A.. (2015). Lower-extremity muscle atrophy and fat infiltration after chronic spinal cord injury. J. Musculoskel. Neuron. 15, 32–41. 25730650 PMC5092153

[B84] Nash M. S. DeGroot J. Martinez-Arizala A. Mendez A. J. (2005). Evidence for an exaggerated postprandial lipemia in chronic paraplegia. J. Spinal Cord Med. 28, 320–325. 10.1080/10790268.2005.11753827 16396382 PMC1864900

[B85] Nash M. S. Groah S. L. Gater D. R. Dyson-Hudson T. A. Lieberman J. A. Myers J.. (2019). Identification and management of cardiometabolic risk after spinal cord injury: clinical practice guideline for health care providers. J. Spinal Cord Med. 1–35. 10.1080/10790268.2018.1511401 30459501 PMC6241225

[B86] Nash M. S. Jacobs P. L. Mendez A. J. Goldberg R. B. (2001). Circuit resistance training improves the atherogenic lipid profiles of persons with chronic paraplegia. J. Spinal Cord Med. 24, 2–9. 10.1080/10790268.2001.11753548 11587430

[B87] Nash M. S. Jacobs P. L. Woods J. M. Clark J. E. Pray T. A. Pumarejo A. E. (2002). A comparison of 2 circuit exercise training techniques for eliciting matched metabolic responses in persons with paraplegia. Arch. Phys. Med. Rehabil. 83, 201–219. 10.1053/apmr.2002.28011 11833023

[B88] Nash M. S. van de Ven I. van Elk N. Johnson B. M. (2007). Effects of circuit resistance training on fitness attributes and upper-extremity pain in middle-aged men with paraplegia. Arch. Phys. Med. Rehabil. 88, 70–75. 10.1016/j.apmr.2006.10.003 17207678

[B89] Nightingale T. E. Eginyan G. Balthazaar S. J. T. Williams A. M. M. Lam T. Krassioukov A. V. (2021). Accidental boosting in an individual with tetraplegia - considerations for the interpretation of cardiopulmonary exercise testing. J. Spinal Cord Med. 1–6. 10.1080/10790268.2020.1871253 33513073 PMC9661994

[B90] Nightingale T. E. Metcalfe R. S. Vollaard N. B. Bilzon J. L. (2017a). Exercise guidelines to promote cardiometabolic health in spinal cord injured humans: time to raise the intensity? Arch. Phys. Med. Rehabil. 98, 1693–1704. 10.1016/j.apmr.2016.12.008 28089898

[B91] Nightingale T. E. Williams S. Thompson D. Bilzon J. L. J. (2017b). Energy balance components in persons with paraplegia: daily variation and appropriate measurement duration. Int. J. Behav. Nutr. Phy.14:132. 10.1186/s12966-017-0590-z 28950900 PMC5615439

[B92] Palmer J. P. Henry D. P. Benson J. W. Johnson D. G. Ensinck J. W. (1976). Glucagon response to hypoglycemia in sympathectomized man. J. Clin. Invest. 57, 522–525. 10.1172/JCI108305 1254731 PMC436678

[B93] Parker K. Uddin R. Ridgers N. D. Brown H. Veitch J. Salmon J.. (2021). The use of digital platforms for adults' and adolescents' physical activity during the COVID-19 pandemic (our life at home): survey study. J. Med. Internet Res. 23:e23389. 10.2196/23389 33481759 PMC7857525

[B94] Piercy K. L. Troiano R. P. Ballard R. M. Carlson S. A. Fulton J. E. Galuska D. A.. (2018). The physical activity guidelines for Americans. Jama 320, 2020–2028. 10.1001/jama.2018.14854 30418471 PMC9582631

[B95] Popok D. W. West C. R. Hubli M. Currie K. D. Krassioukov A. V. (2017). Characterizing the severity of autonomic cardiovascular dysfunction after spinal cord injury using a novel 24 hour ambulatory blood pressure analysis software. J. Neurotraum. 34, 559–566. 10.1089/neu.2016.4573 27573583

[B96] Richardson E. V. Smith B. Papathomas A. (2017). Collective stories of exercise: Making sense of gym experiences with disabled peers. Adapt. Phys. Activ. Q. 34, 276–294. 10.1123/apaq.2016-0126 28727508

[B97] Rimmer J. H. Mehta T. Wilroy J. Lai B. Young H. J. Kim Y.. (2019). Rationale and design of a Scale-Up Project Evaluating Responsiveness to Home Exercise And Lifestyle Tele-Health (SUPER-HEALTH) in people with physical/mobility disabilities: a type 1 hybrid design effectiveness trial. BMJ Open 9:e023538. 10.1136/bmjopen-2018-023538 30928927 PMC6475257

[B98] Rimmer J. H. Rubin S. S. Braddock D. (2000). Barriers to exercise in African American women with physical disabilities. Arch. Phys. Med. Rehabil. 81, 182–188. 10.1016/S0003-9993(00)90138-2 10668772

[B99] Scelza W. M. Kalpakjian C. Z. Zemper E. D. Tate D. G. (2005). Perceived barriers to exercise in people with spinal cord injury. Arch. Phys. Med. Rehabil. 84, 576–583. 10.1097/01.phm.0000171172.96290.67 16034226

[B100] Segal J. L. Thompson J. F. Tayek J. A. (2007). Effects of long-term 4-aminopyridine therapy on glucose tolerance and glucokinetics in patients with spinal cord injury. Pharmacotherapy 27, 789–792. 10.1592/phco.27.6.789 17542761

[B101] Simmons O. L. Kressler J. Nash M. S. (2014). Reference fitness values in the untrained spinal cord injury population. Arch. Phys. Med. Rehabil. 95, 2272–2278. 10.1016/j.apmr.2014.06.015 25007709

[B102] Solinsky R. Mercier H. Picard G. Taylor J. A. (2020). Cardiometabolic effects of high-intensity hybrid functional electrical stimulation exercise after spinal cord injury. PM R. 10.1002/pmrj.12507. [Epub ahead of print]. 33027550 PMC9284583

[B103] Taylor J. L. Holland D. J. Spathis J. G. Beetham K. S. Wisloff U. Keating S. E.. (2019). Guidelines for the delivery and monitoring of high intensity interval training in clinical populations. Prog. Cardiovasc. Dis. 62, 140–146. 10.1016/j.pcad.2019.01.004 30685470

[B104] Vives Alvarado J. R. Felix E. R. Gater D. R. Jr. (2021). Upper extremity overuse injuries and obesity after spinal cord injury. Top. Spinal Cord Inj. Rehabil. 27, 68–74. 10.46292/sci20-00061 33814884 PMC7983631

[B105] Wang Y. H. Chen S. Y. Wang T. D. Hwang B. S. Huang T. S. Su T. C. (2009). The relationships among serum glucose, albumin concentrations and carotid atherosclerosis in men with spinal cord injury. Atherosclerosis 206, 528–534. 10.1016/j.atherosclerosis.2009.02.035 19349048

[B106] West C. R. Popok D. Crawford M. A. Krassioukov A. V. (2015). Characterizing the temporal development of cardiovascular dysfunction in response to spinal cord injury. J. Neurotraum. 32, 922–930. 10.1089/neu.2014.3722 25630034

[B107] West C. R. Romer L. M. Krassioukov A. (2013). Autonomic function and exercise performance in elite athletes with cervical spinal cord injury. Med. Sci. Sports Exerc. 45, 261–267. 10.1249/MSS.0b013e31826f5099 22914247

[B108] Weston K. S. Wisloff U. Coombes J. S. (2014). High-intensity interval training in patients with lifestyle-induced cardiometabolic disease: a systematic review and meta-analysis. Br. J. Sports Med. 48, 1227–U52. 10.1136/bjsports-2013-092576 24144531

[B109] Wilroy J. Mehta T. Pekmezi D. Thirumalai M. Young H.-J. Rimmer J. (2017). Scale Up Project Evaluating Responsiveness to Home Exercise And Lifestyle Tele-Health (SUPER HEALTH). Arch. Phys. Med. Rehabil. 98:e107. 10.1016/j.apmr.2017.08.345 30928927 PMC6475257

[B110] Wilroy J. D. Lai B. Davlyatov G. Mehta T. Thirumalai M. Rimmer J. H. (2020). Correlates of adherence in a home-based, self-managed exercise program tailored to wheelchair users with spinal cord injury. Spinal Cord 1–8. 10.1038/s41393-020-0526-3 32541883 PMC7962257

[B111] Yarar-Fisher C. Bickel C. S. Windham S. T. McLain A. B. Bamman M. M. (2013). Skeletal muscle signaling associated with impaired glucose tolerance in spinal cord-injured men and the effects of contractile activity. J. Appl. Physiol. 115, 756–764. 10.1152/japplphysiol.00122.2013 23766505 PMC4073980

[B112] Zleik N. Weaver F. Harmon R. L. Le B. Radhakrishnan R. Jirau-Rosaly W. D.. (2019). Prevention and management of osteoporosis and osteoporotic fractures in persons with a spinal cord injury or disorder: a systematic scoping review. J. Spinal Cord Med. 42, 735–759. 10.1080/10790268.2018.1469808 29745791 PMC6830234

[B113] Zlotolow S. P. Levy E. Bauman W. A. (1992). The serum lipoprotein profile in veterans with paraplegia: the relationship to nutritional factors and body mass index. J. Am. Paraplegia Soc. 15, 158–162. 10.1080/01952307.1992.11735869 1500941

